# Particle-associated bacteria differentially influence the aggregation of the marine diatom *Minutocellus polymorphus*

**DOI:** 10.1038/s43705-022-00146-z

**Published:** 2022-08-18

**Authors:** Bianca N. Cruz, Susanne Neuer

**Affiliations:** 1 School of Life Sciences, Arizona State University, Tempe, AZ USA; 2 Center for Fundamental and Applied Microbiomics, Biodesign Institute, Arizona State University, Tempe, AZ USA

**Keywords:** Water microbiology, Environmental sciences

## Abstract

The aggregation of phytoplankton leads to the settling of particulate organic carbon in the form of marine snow, making it an important process in marine biogeochemical cycles. Diatoms >20 µm in size are considered to contribute appreciably to sinking particle fluxes due to aggregation and the production of transparent exopolymeric particles (TEP), the matrix for marine snow aggregates; however, it is not known whether nano-sized (2–20 µm) diatoms are able to aggregate and produce TEP. Here, we tested the aggregation and production of TEP by the nano-diatom *Minutocellus polymorphus* and investigated if interactions with bacteria influence aggregation by comparing axenic *M. polymorphus* cultures with co-cultures of the diatom with bacterial taxa known to colonize marine snow particles. We found that *M. polymorphus* form sinking aggregates and produce TEP comparably to other phytoplankton groups and that aggregation and TEP production were influenced depending on the species of bacteria added. Aggregation was enhanced in the presence of *Marinobacter adhaerens* HP15, but not in the presence of *Pseudoalteromonas carrageenovora* or *Vibrio thalassae*. Cell aggregation mediated by interactions with specific bacterial species are possible mechanisms behind the export of nano-sized diatoms, such as *M. polymorphus*, especially in oligotrophic open ocean regions where small phytoplankton dominate.

## Introduction

Diatoms are an abundant and ubiquitous class of phytoplankton responsible for contributing up to 40% of the ocean’s primary productivity [1]. Their blooms are often followed by aggregation and subsequent sedimentation events that account for ~40% of the total export of particulate organic carbon (POC) [2], thus, they are considered significant contributors to export fluxes and global biogeochemical cycles. In biogeochemical models, diatoms are frequently considered to be micro-phytoplankton (>20 µm), but, many ubiquitous species overlap with the nano- (2–20 µm) and picoplanktonic (≤2 µm) size-classes [3]. Due to their minute size, nano- and pico-sized diatoms are typically considered to contribute negligibly to particle fluxes [4]; however, there is increasing evidence against this notion [5–7]. Observations of abundant frustules belonging to *Minidiscus trioculatus* (ca. 2 µm) in sediment traps deployed in the Northwestern Mediterranean at 2400 m [8] as well as the consistent representation of *M. trioculatus* and *Minutocellus polymorphus* (ca. 4–6 µm) in size-fractionated water samples (≥180 µm) collected at 700 m depth [9] suggest high sinking velocities as a result of their incorporation into sinking particles and/or aggregation mechanisms. Furthermore, DNA-based analyses of particle trap material from the Sargasso Sea have demonstrated significant overrepresentation [5] and specificity [10] of these small diatom genera in sinking particles compared to the ambient seawater, further suggesting an underestimation of their role in particle fluxes, especially in open ocean regions, where small phytoplankton are most abundant [11].

Hypothesized mechanisms of export of pico- and nanophytoplankton include aggregation and subsequent increase of their effective size [7, 12] which is dependent on the abundance, size, sticking efficiency, and relative sinking velocities of surrounding particles [13]. Particle sticking efficiency can be a result of cell surface properties [13] and/or cellular exudates [14, 15] including exopolysaccharides such as the transparent exopolymeric particles (TEP). TEP further facilitate aggregation and subsequent settling of cells during phytoplankton blooms and are a significant component of the matrix of marine snow aggregates [16, 17]. Phytoplankton are considered the main producers of TEP [18], and the presence of bacteria has been shown to increase this production [19–22], suggesting interactions that may have significant implications in aggregation and enhanced sinking rates of senescent phytoplankton. In addition to enhancing the production of TEP by phytoplankton, some bacteria can produce TEP [23], as well as consume and/or modify its precursors [24, 25]. As a result, bacteria-mediated aggregation in phytoplankton can vary with the ambient bacterial species.

To explain the observations suggesting a significant contribution of nano-sized diatom cells to sinking particles and their export fluxes, controlled laboratory experiments are necessary to test the potential mechanisms behind the export of these abundant, yet often-overlooked, diatom genera. In this study, our objectives were: (1) to investigate whether the marine nano-diatom *Minutocellus polymorphus* forms aggregates and produces TEP and (2) to determine if the presence of distinct marine bacteria distinct marine bacteria enhance the aggregation of *M. polymorphus*. We chose a two-phase approach with a first set of experiments focusing on the growth phase of diatoms and the second set targeting aggregation and sedimentation at the end of a bloom (Table 1). Experiments investigated the diatom *M. polymorphus* in combination with one of three known particle-associated bacteria [10, 26]. We hypothesized that (1) *M. polymorphus* forms fast-sinking aggregates within a matrix of TEP, and (2) that aggregation and TEP production by *M. polymorphus* is enhanced in the presence of bacteria.

**Table 1:** Outline of the experiments performed in this study.

Transparent exopolymeric particle production and micro-aggregate formation Bloom				
	Diatom-only		Diatom + Bacteria	
Growth experiment 1	*M. polymorphus*		*M. polymorphus* + *M. adhaerens*	
Growth experiment 2	*M. polymorphus*		*M. polymorphus* + *V. thalassae*	
			*M. polymorphus* + *P. carrageenovora*	
**Sinking aggregate formation in roller tanks** **End of bloom and sedimentation**				
	**Diatom-only**	**Bacteria-only**	**Diatom** + **Bacteria**	**+ 2.5** **µm beads**
Lower cell abundance (ca. 10^3^ cells mL^−1^)	*M. polymorphus*	*M. adhaerens* *V. thalassae* *P. carrageenovora*	*M. polymorphus* + *M. adhaerens* *M. polymorphus* + *V. thalassae* *M. polymorphus* *+ P. carrageenovora*	*M. polymorphus* + beads *M. adhaerens* + beads *V. thalassae* + beads *P. carrageenovora* + beads
Higher cell abundance (ca. 10^5^ cells mL^−1^)	*M. polymorphus*	*M. adhaerens*	*M. polymorphus* + *M. adhaerens*	N.A.

*Minutocellus polymorphus* were incubated with or without the addition of bacteria in flasks and sampled throughout their growth (representative of bloom conditions), to determine the production of transparent exopolymeric particles (TEP) and the formation of micro-aggregates, and in roller tanks to investigate the formation of sinking aggregates (representative of end of bloom conditions). The growth experiment with the addition of *Marinobacter adhaerens* (growth experiment 1) was carried out independently from that with the addition of *Pseudoalteromonas carrageenovora* and *Vibrio thalassae* (growth experiment 2). Roller tank experiments were performed with total cell numbers of 10^3^ cells mL^−1^ (lower abundance) and 10^5^ cells mL^−1^ (higher abundance).

## Methods

### Diatoms and bacterial isolates

Stock cultures of marine *Minutocellus polymorphus* [27] (CCMP497, National Center for Marine Algae and Microbiota, NCMA) were maintained in L1 medium [28] prepared in artificial seawater [29] (salinity 35) and incubated in an environmental growth chamber (Conviron) at 23 ± 1 °C with a light intensity of 65–80 µmol photons  m^−2^ s^−1^ in a 14 h:10 h light-dark cycle. Experiments were performed with *M. polymorphus* due to the unavailability of axenic *Minidiscus* spp. cultures. Stock cultures of *Vibrio thalassae* (DSM102810, DSMZ-German Collection of Microorganisms and Cell Cultures GmbH), *Pseudoalteromonas carrageenovora* (DSM6820, DSMZ), and *Marinobacter adhaerens* HP15 [26] were maintained on Marine Agar (BD Difco 2216, Becton Dickinson, NJ; ZoBell, 1941) plates at 23 ± 1 °C. Bacteria pre-cultures were grown in Marine Broth (BD Difco 2216, Becton Dickinson, NJ; ZoBell, 1941) on a shaker at 150 RPM for 48 h and washed three times with and resuspended in sterile L1 medium to minimize carry-over of nutrients or bacterial-derived DOM into experimental co-cultures. Bacterial cells were washed by centrifugation to form a pellet at 3220 × *g* for 7 min at 22 °C.

### TEP and micro-aggregate formation during growth

#### Experimental conditions

Treatments consisted of triplicate cultures of axenic *M. polymorphus* as a bacteria-free negative control treatment and *M. polymorphus* in co-culture with each of the three bacterial species (Table 1). The co-culture experiment with *M. adhaerens* (growth experiment 1) was performed independently from that with *V. thalassae* and *P. carrageenovora* (growth experiment 2), each with their own triplicate negative control treatments of axenic *M. polymorphus* (Table 1). Exponentially growing *M. polymorphus* cells were used to inoculate all treatments (ca. 10^5^ cells mL^−1^). Bacterial cell concentrations were about an order of magnitude higher than diatom concentrations. Experimental cultures were grown in the same conditions as the stock cultures. 100 mL samples were taken every other day and immediately used to determine the volume of micro-aggregates in cultures as described below. Samples were then preserved in glutaraldehyde (1% [v/v] final concentration, Sigma-Aldrich) for TEP measurements and for the quantification of single-cell abundances in cultures. All axenic treatments were tested for bacterial and fungal contamination prior to experiment inoculation and at the end of all experimental periods by inoculation in L1 medium with added peptone and methylamine-HCl, as suggested by the NCMA. Axenic *M. polymorphus* treatments were bacteria-free throughout the experiments. Co-culture treatments were tested for contamination of non-co-cultured bacteria at every sampling period by epifluorescence microscopic observation of cell morphologies, as well as colony morphologies by streaking onto Marine Agar (BD Difco 2216, Becton Dickinson, NJ; ZoBell 1941) plates at days 1, 10, and following the end of the experimental periods.

#### Quantification of cell abundance and suspended micro-aggregates

Cell abundances in the cultures were determined with the use of epifluorescence microscopy (Carl Zeiss AxioScope.A1). Glutaraldehyde-fixed samples were stained with the nucleic acid dye DAPI (4′,6-diamidino-2-phenylindole, 0.03 M, Sigma-Aldrich), and filtered onto gray 0.2 µm pore-size polycarbonate membranes (GVS Life Technologies, ME). Chlorophyll-*a* emission by *M. polymorphus* cells and DAPI-stained bacteria were visualized under 450–490 nm excitation and 380–400 nm, respectively.

Volume concentrations of micro-aggregates “suspended” in cultures (i.e., non-sinking particles with an equivalent spherical diameter [ESD] of 5–60 µm) were determined at every sampling period using a Multisizer 3 Particle Counter (Beckman Coulter, CA). Prior to fixation with glutaraldehyde, duplicate samples were diluted to a 1–10% final particle concentration with Isoton II diluent (Beckman Coulter, CA) and aggregates were measured and quantified with a 100 µm aperture tube. The volume concentration of aggregates was calculated in µm^3^ per mL.

#### TEP

TEP concentrations in the co-cultures and axenic cultures were determined as described by Passow and Alldredge [30]. 10 mL of glutaraldehyde-fixed culture samples were filtered through duplicate 0.4 µm pore-size polycarbonate membranes (GVS Life Technologies, ME) at a low and constant vacuum pressure (100 mm Hg). The retained TEP was subsequently stained with 0.5 mL of the acidic polysaccharide-specific Alcian Blue (AB) dye (8GX, Sigma-Aldrich), followed by a 0.5 mL rinse with MilliQ water for the removal of excess stain and stored at −40 °C until analysis. Prior to staining, the pre-calibrated [31] 0.02% (w/v) AB working solution pH-adjusted with 0.06% (v/v) acetic acid (final pH 2.5) was passed through a 0.2 µm Acrodisc syringe filter (Pall Corporation, NY) to remove the undissolved dye. Membranes were soaked in 6 mL of 80% (v/v) sulfuric acid for 3 h to extract the AB-stained TEP and absorption was then measured using a spectrophotometer (Shimadzu UV-1900i, Shimadzu, Kyoto, JP) at 787 nm. Duplicate stained filters with sterile media functioned as blanks. TEP concentrations were calculated using a calibration factor of the AB dye determined with xanthan gum (f-factors: 81.70 for experiments with *M. adhaerens* and 83.83 for experiments with *V. thalassae* and *P. carrageenovora*) and expressed in µg of xanthan gum equivalent units (µg XG eq.) as described by Bittar et al. [31]. To compare TEP concentrations between treatments and with other phytoplankton groups, concentrations were normalized to diatom cell abundances and biovolumes, respectively. TEP production rates were calculated as previously described [32].

### Aggregation in roller tanks

To investigate the formation of visible aggregates by *M. polymorphus* with and without the presence of bacteria, diatom and bacteria co-cultures were incubated in roller tanks that simulate the natural collision of particles as they would occur in situ [33]. *M. polymorphus* pre-cultures were incubated until the late exponential phase of growth was achieved (ca. 10 days). Bacteria were grown overnight in Marine Broth prior to being washed three times in artificial seawater. Bacteria were then incubated in artificial seawater for 24 hours prior to inoculation within roller tanks to minimize nutrient carry-over. *M. polymorphus* pre-cultures were diluted to cell abundances simulating spring conditions in the Sargasso Sea as observed by Cruz and Neuer [20] (ca. 10^3^ cells mL^−1^, hereafter termed “lower cell abundance experiments”; Table 1) and incubated in cylindrical 1.25 L Plexiglass roller tanks [33] with artificial seawater [29]. Axenic *M. polymorphus* were inoculated at a final cell concentration of 10^3^ cells mL^−1^ and co-culture treatments were inoculated with *M. polymorphus* cells at 10^2^ cells mL^−1^ and bacteria at 10^3^ cells mL^−1^. Axenic bacterial treatments were inoculated at a final concentration of 10^3^ cells mL^−1^ (Table 1). Control treatments consisted of the following: axenic *M. polymorphus*, bacteria-only (*V. thalassae, P. carrageenovora*, or *M. adhaerens*), and the addition of sterile silica microspheres (2.5 µm, 1.4 × 10^9^ beads mL^−1^ stock concentration; Bangs Laboratories, IN) to axenic *M. polymorphus* cell suspensions (final concentrations: 10^2 ^*M. polymorphus* cells mL^−1^; 10^3^ beads mL^−1^) and to bacteria-only cell suspensions (final concentrations: 10^2^ beads mL^−1^ and 10^3^ bacterial cells mL^−1^). An experiment with higher total cell abundances (ca. 10^5^ cells mL^−1^, hereafter termed “higher cell abundance experiments”; Table 1) was performed with the addition of *M. adhaerens*, composed of axenic treatments with *M. polymorphus* or bacteria and treatments with *M. polymorphus* and the addition of *M. adhaerens* (Table 1). Duplicate tanks for each treatment were rotated on a rolling platform at 3.5 rotations per minute in the dark at 24 °C for 7 days and checked daily for aggregate formation. At the end of the incubation period, the number of visible aggregates (ca. > 0.5 mm) formed in each tank was counted and photographs of aggregates were taken within the roller tanks. The ESD of each imaged particle was then determined using ImageJ image analysis software (http://rsb.info.nih.gov/ij/). To determine sinking velocities [34], aggregates from each roller tank were gently transferred with a wide-bore pipette into a 1 L graduated cylinder and released at 1 cm under the air-water interface. The cylinder was filled with artificial seawater at the same salinity and temperature as roller tanks, and the settling time of each aggregate was determined through a vertical distance of 32.6 cm. Settling times were subsequently converted to velocities in meters per day.

### Statistical analyses

Data are presented as the means of triplicate cultures with standard deviations. Two-sample Student’s *t*-tests were performed to check for statistically significant differences in the formation of micro-aggregates and the production of TEP between axenic and co-culture treatments in growth experiments 1 and 2. Kruskal-Wallis corrected for multiple comparisons with a post-hoc Dunn’s test were used to test for significant differences in the formation of sinking aggregates between treatments in the roller tank experiments. Statistical analyses were performed using R [35] software.

### Data deposition

All experimental data are as well as their associated metadata are available through the Biological and Chemical Oceanography Data Management Office (https://bco-dmo.org/dataset/876461).

## Results

### TEP and micro-aggregate formation during growth

#### Cell growth

**Fig. 1:**
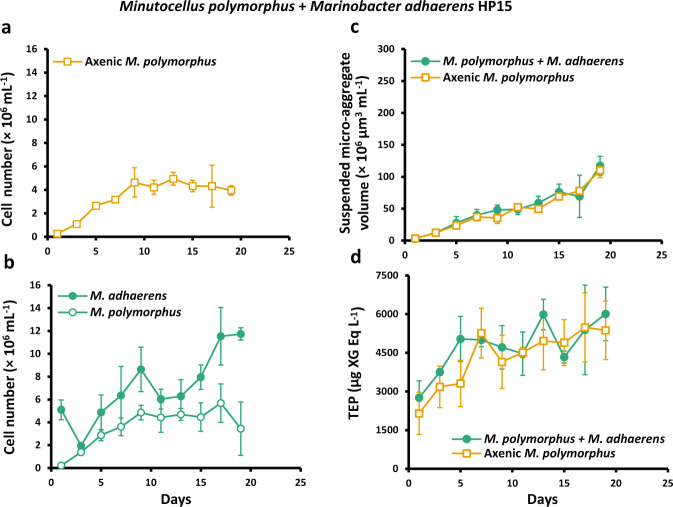
Cell growth, TEP concentration, and micro-aggregate (5–60 μm) volume concentration in *Minutocellus polymorphus* co-cultures with *Marinobacter adhaerens* HP15. Diatom cell concentration in growth experiment 1 with axenic *Minutocellus polymorphus* (**a**) and diatom and bacteria cell concentration in co-culture treatments with *M. polymorphus* and the addition of *Marinobacter adhaerens* HP15 (**b**), as well as total volume of suspended micro-aggregates (**c**) and transparent exopolymeric particle (TEP) concentrations (**d**) in all treatments. Symbols and error bars denote the means and standard deviations of triplicate treatments.

**Fig. 2:**
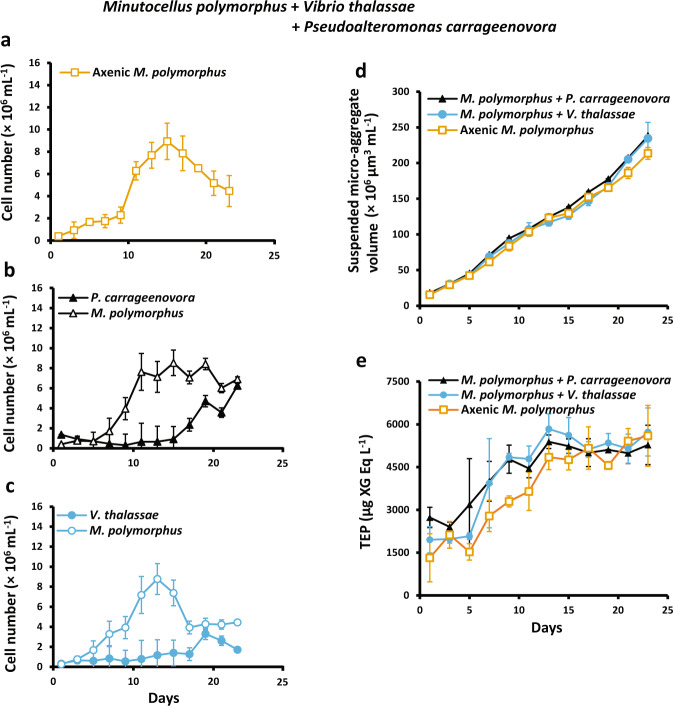
Cell growth, TEP concentration, and micro-aggregate (5–60 μm) volume concentration in *Minutocellus polymorphus* co-cultures with *Vibrio thalassae* and *Pseudoalteromonas carrageenovora*. Diatom cell concentration in growth experiment 2 with axenic *Minutocellus polymorphus* (**a**) and diatom and bacteria cell concentration in co-culture treatments with *M. polymorphus* and the addition of *Pseudoalteromonas carrageenovora* (**b**) or *Vibrio thalassae* (**c**), as well as total volume of suspended micro-aggregates (**d**) and transparent exopolymeric particle (TEP) concentrations (**e**) in all treatments. Symbols and error bars denote the means and standard deviations of triplicate treatments.

**Fig. 3:**
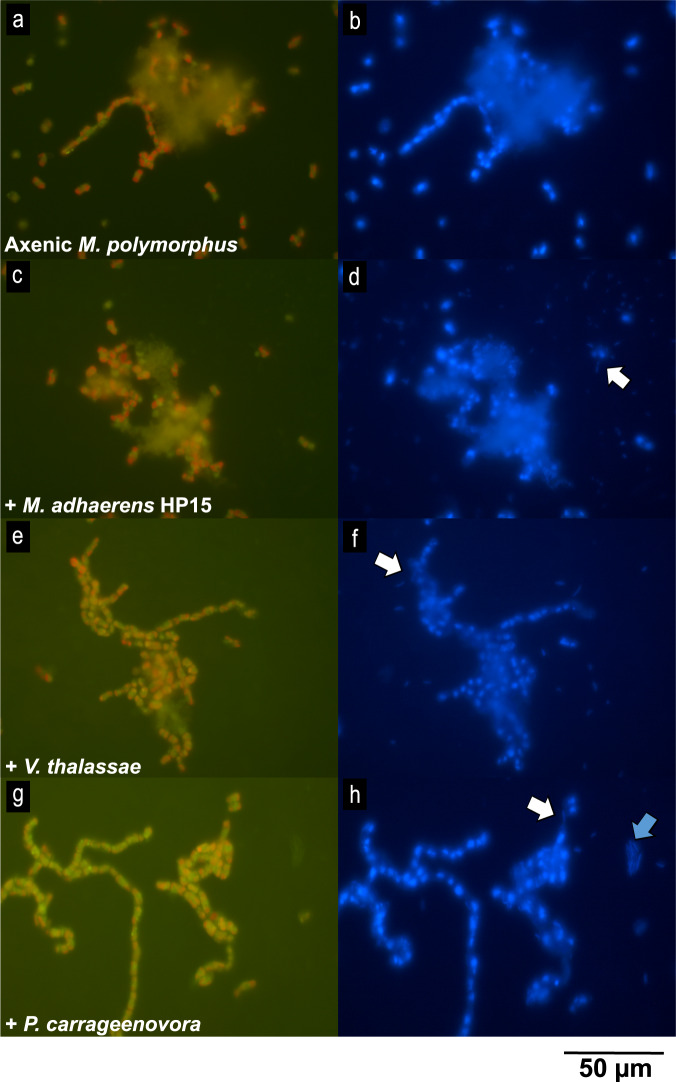
Microscopy images of microaggregates and attached bacteria. Epifluorescence microscopy images of axenic *Minutocellus polymorphus* (**a**, **b**) and co-cultures of *M. polymorphus* and *Marinobacter adhaerens* HP15 (**c**, **d**), *Vibrio thalassae* (**e**, **f**), and *Pseudoalteromonas carrageenovora* (**g**, **h**) under blue (**a**, **c**, **e**, **g**) and UV (**b**, **d**, **f**, **h**) excitation wavelengths. Images were taken during the exponential phases of growth. Red: chlorophyll-*a* autofluorescence, blue: DAPI-bound nucleic acids. White arrows: diatom-attached bacteria; blue arrows: bacterial aggregates. Amorphous material is considered TEP.

*M. polymorphus* cells grew for the first 9–11 days of incubation in axenic and co-culture treatments, with maximum growth rates of 0.6 ± 0.02 d^−1^ (days 1–5, means ± standard deviations of *n* = 3 cultures; Figs. 1a, b and 2a–c). There were no differences in the growth rates of *M. polymorphus* with and without the presence of bacteria (*t*-test, *p* > 0.05 for all). *M. adhaerens* HP15 cells declined within the first 2 days of incubation (days 1–3, −0.5 ± 0.1 d^−1^; Fig. 1b) which coincided with the period of highest growth of the co-cultured *M. polymorphus* (days 1–5; Fig. 1b), followed by a rapid increase (*M. adhaerens*: days 3–5, 0.4 ± 0.2 d^−1^); however, *M. adhaerens* declined once again (days 9–11, −0.2 ± 0.2 d^−1^) during *M. polymorphus’* stationary phase (days 9–17, 0.02 ± 0.02 d^−1^), followed by a final increase towards the end of the experimental period (days 13–17, 0.1 ± 0.03 d^−1^). Cell numbers of *P. carrageenovora* declined during the first 6 days of the experiment (days 1–7, −0.09 ± 0.1 d^−1^; Fig. 2b), and did not grow for 4 days (days 7–11, 2.5 × 10^−3^ ± 0.1 d^−1^) until the stationary phase of the co-cultured *M. polymorphus* (days 11–19, 0.02 ± 0.01 d^−1^; Fig. 2b), which was then followed by an increase in the cell abundance of *P. carrageenovora* (days 13–23, 0.2 ± 0.03 d^−1^). *V. thalassae* cells grew for the first 2 days (days 1–3, 0.3 ± 0.1 d^−1^; Fig. 2c), followed by no growth (days 3–17, −0.05 ± 0.03 d^−1^) until the decline phase of the co-cultured *M. polymorphus* (*V. thalassae*: days 15–23, 0.2 ± 0.04 d^−1^, *M. polymorphus*: days 15–23, −0.06 ± 0.02; Fig. 2c). Microscopy observations of the co-cultures demonstrated that all tested bacterial isolates attached to single cells, chains, and aggregates of *M. polymorphus* (Fig. 3). *P. carrageenovora*-only aggregates were observed in co-cultures throughout the experimental period (Fig. 3h).

#### Suspended micro-aggregates

The total volume concentration of suspended (5–60 µm) micro-aggregates increased throughout the incubation periods in both axenic and co-culture treatments (Figs. 1c and 2d), with peak concentrations occurring at the end of the experiments. Maximum suspended micro-aggregate volume concentrations in axenic *M. polymorphus* cultures were 110 ± 11 × 10^6^ µm^3^ mL^−1^ for experiments with *M. adhaerens* (Fig. 1c) and twice that for experiments with *V. thalassae* and *P. carrageenovora* (Fig. 2d). Maximum volume concentrations were 117 ± 15 ×  10^6^ µm^3^ mL^−1^ in co-cultures with *M. adhaerens* (Fig. 1c), 234 ± 23 with *V. thalassae* (Fig. 2d), and 238 ± 8 × 10^6^ µm^3^ mL^−1^ with *P. carrageenovora* (Fig. 2d). There were no differences in the volume concentration of suspended micro-aggregates between axenic *M. polymorphus* cultures and co-cultures during the exponential phases of diatom growth (Figs. 1c and 2d; Table 2). However, compared to axenic cultures, volume concentrations were significantly higher in co-cultures with *M. adhaerens* during the stationary growth periods of the co-cultured *M. polymorphus* (*t*-test, *p* = 0.04; Table 2), and in co-cultures with *P. carrageenovora* during the stationary and decline periods of *M. polymorphus* (*t*-test, stationary: *p* = 0.03, decline: *p* = 0.006; Table 2). Volume concentrations were significantly higher in co-cultures with *V. thalassae* at day 21 (186 ± 8 × 10^6^ µm^3^ mL^−1^ axenic *M. polymorphus*, 205 ± 2 × 10^6^ µm^3^ mL^−1^ co-cultures with *V. thalassae*, *t*-test, *p* = 0.01), followed by a continuous increase to day 23 (Fig. 2d); however, due to a high variance between the triplicate co-cultures, suspended micro-aggregate volume concentrations were not statistically different from axenic cultures at day 23, leading to non-significant differences when considering the entire decline period (days 19–23, *t-*test, *p* = 0.62; Table 2).

**Table 2:** Transparent exopolymeric particle (TEP) production, TEP concentration normalized to diatom cell abundance, and volume of suspended micro-aggregates during the exponential, stationary, and decline growth phases of *Minutocellus polymorphus* in axenic cultures and in co-cultures with *Marinobacter adhaerens* HP15, *Vibrio thalassae*, or *Pseudoalteromonas carrageenovora*.

Transparent exopolymeric particle production and micro-aggregate formation Bloom				
Treatment	Growth phase	TEP production (d^−1^)	Diatom cell-normalized TEP concentration (×10^−6^ µg XG eq. cell^−1^)	Micro-aggregate volume concentration (×10^6^ µm^3^ mL^−1^)
*M. polymorphus*, axenic (Growth experiment 1)	Exponential (1–9)	0.09 ± 0.05	2.96 ± 2.97	22 ± 13
	Stationary (9–13)	0.05 ± 0.09	1.02 ± 0.29	45 ± 9
	Decline (13–19)	0.01 ± 0.02	1.20 ± 0.31	76 ± 23
*M. polymorphus*, axenic (Growth experiment 2)	Exponential (1–11)	0.11 ± 0.08	2.0 ± 1.8	56 ± 32
	Stationary (11–15)	0.07 ± 0.03	0.60 ± 0.12	119 ± 12
	Decline (15–23)	0.02 ± 0.02	0.87 ± 0.35	169 ± 30
*M. polymorphus* + *M. adhaerens*	Exponential (1–9)	0.07 ± 0.03	3.84 ± 4.42	26 ± 18
	Stationary (9–17)	0.01 ± 0.06	1.07 ± 0.29	**60** ± **9**
	Decline (17–19)	0.07 ± 0.09	1.20 ± 0.57	93 ± 34
*M. polymorphus* + *V. thalassae*	Exponential (1–11)	0.09 ± 0.02	2.69 ± 2.54	59 ± 33
	Stationary (11–15)	0.04 ± 0.04	0.71 ± 0.12	117 ± 10
*M. polymorphus* + *P. carrageenovora*	Exponential (1–11)	0.07 ± 0.05	**3.57** ± **2.50**	61 ± 34
	Stationary (11–19)	**0.02** ± **0.009**	0.67 ± 0.09	**141** ± **27**
	Decline (19–23)	0.007 ± 0.03	**0.70** ± **0.06**	**207** ± **28**
	Decline (15–23)	0.002 ± 0.03	**1.17** ± **0.25**	176 ± 42

Note that the experiment with *M. adhaerens* was carried out independently from *V. thalassae* and *P. carrageenovora*, both with their own negative control treatment of axenic *M. polymorphus*. Values in parentheses are the experimental days affiliated with each corresponding growth phase and those in bold are significantly different to their axenic counterparts. Values are the means ± standard deviations of triplicate treatments.

#### TEP

TEP accumulated in co-cultures and axenic treatments as the abundance of *M. polymorphus* cells increased (Figs. 1d and 2e). In axenic *M. polymorphus* cultures, most of the production of TEP occurred during periods of exponential growth (Table 2), with concentrations increasing from 2.1 ± 0.8 µg XG eq. mL^−1^ on day 1 to 4.1 ± 1 µg XG eq. mL^−1^ on day 9 for experiments with *M. adhaerens* (Fig. 1d). In experiments with *V. thalassae* and *P. carrageenovora*, TEP concentrations in axenic *M. polymorphus* cultures increased from 1.3 ± 0.8 µg XG eq. mL^−1^ on day 1 to 3.6 ± 0.6 µg XG eq. mL^−1^ on day 11 (Fig. 2e). To compare between treatments with differing cell abundances, we normalized TEP concentrations to diatom cell numbers. Maximum cell-normalized TEP concentrations in axenic *M. polymorphus* and in co-cultures occurred during the exponential phase (Table 2). The addition of *V. thalassae* led to higher cell-normalized TEP concentrations during the decline phase of the co-cultured *M. polymorphus* (*t-*test, *p* = 0.01; Table 2, but not in the exponential (*p* = 0.4) or stationary (*p* = 0.07) phases. There were no differences in TEP production rates between axenic treatments and co-cultures with *V. thalassae* (*t*-test, *p* > 0.05 for all growth phases; Table 2). We also found no significant differences in the TEP production rates or cell-normalized TEP concentrations between axenic cultures and co-cultures with *M. adhaerens* (*t-*test, *p* > 0.05 for all growth phases; Table 2). Compared to axenic treatments, cell-normalized TEP concentrations were significantly higher in co-cultures with *P. carrageenovora* during the exponential growth period of the co-cultured *M. polymorphus* (*t*-test, *p* = 0.04; Table 2), but significantly lower during the decline periods of the co-cultured *M. polymorphus* (*t*-test, *p* = 0.04; Table 2). TEP production rates were also significantly lower in co-cultures with *P. carrageenovora* during the stationary phase of *M. polymorphus* (*t*-test, *p* = 0.04; Table 2). These lower TEP production rates and cell-normalized TEP concentrations occurred in parallel to increases in *P. carrageenovora* abundances (Fig. 2b).

### Aggregation in roller tanks

**Fig. 4:**
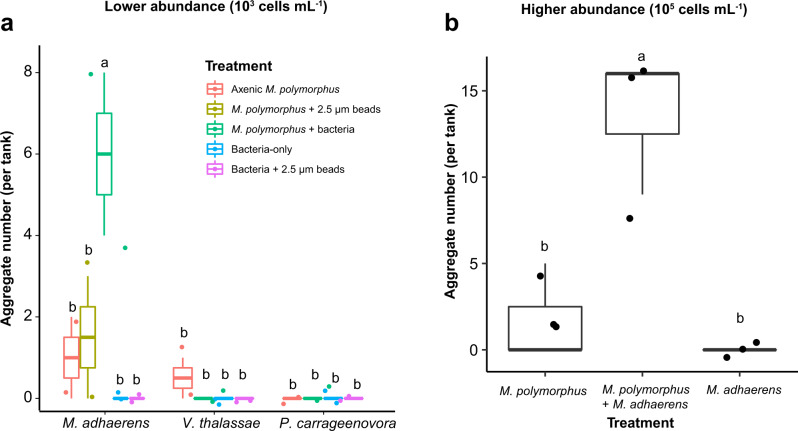
Aggregation of *Minutocellus polymorphus* in roller tanks with and without the addition of *Marinobacter adhaerens* HP15, *Vibrio thalassae*, or *Pseudoalteromonas carrageenovora*. Number of visible sinking aggregates formed in roller tanks with axenic *Minutocellus polymorphus*, and *M. polymorphus* with the addition of 2.5 µm silica beads*, Marinobacter adhaerens* HP15, *Vibrio thalassae*, or *Pseudoalteromonas carrageenovora* in the low cell abundance experiments (**a** ca. 10^3^ cells mL^−1^; Table 3) and of axenic *M. polymorphus* and *M. polymorphus* with the addition of *M. adhaerens* in the high cell abundance experiments (**b** 10^5^ cells mL^−1^; Table 4). Lowercase letters indicate significant differences between treatments.

In addition to quantifying the development of suspended micro-aggregates in batch cultures, we performed roller tank experiments to enhance the formation of visible, sinking aggregates. *M. polymorphus* formed visible aggregates without the addition of bacteria in all experiments, with fewer aggregates (0–2 aggregates) formed in experiments with a lower total cell abundance (10^3^ cells mL^−1^) compared to the experiments containing a higher total cell abundance (0–5 aggregates; 10^5^ cells mL^−1^; Fig. 4; Tables 3 and 4). Axenic *M. polymorphus* aggregates in the higher cell abundance experiments were brown in color, compact, and resilient to handling for the determination of sinking velocities, in contrast to aggregates formed with the addition of *M. adhaerens* and those formed in the lower cell abundance experiments, which were white, amorphous, and much more fragile. Aggregates in the higher cell abundance experiments had ESD’s of 1.7–2.2 mm (*n* = 3 aggregates; Table 4) and sank at velocities of 225–485 m d^−1^ (*n* = 5 aggregates; Table 4). The addition of *M. adhaerens* led to the formation of a significantly higher number of aggregates compared to axenic *M. polymorphus* in both experiments—for instance, in the higher cell abundance experiments, 0–5 aggregates formed in axenic treatments, while 9–16 aggregates formed with the addition of *M. adhaerens* (Kruskal-Wallis and post-hoc Dunn’s test, *p* = 0.04, *n* = 3 tanks; Fig. 4; Tables 3 and 4). No aggregation was observed with the addition of *V. thalassae* or *P. carrageenovora* (Fig. 4a; Table 3). Compared to treatments with axenic *M. polymorphus* and with axenic bacteria, there was no significantly enhanced aggregation with the addition of 2.5 µm silica beads (Kruskal–Wallis and post-hoc Dunn’s test, *p* > 0.05 for all; Fig. 4; Table 3).

**Table 3:** Abundance of aggregates formed in roller tank experiments inoculated to lower total cell abundances (10^3^ cells mL^−1^).

Sinking aggregate formation in roller tanks End of bloom and sedimentation			
Treatment	Background cell abundance (×10^3^ mL^−1^)		Aggregate abundance (per tank)
	Before rotation	After rotation	
*M. polymorphus*, axenic	1.9 ± 0.6	1.6 ± 0.4	0.50 ± 0.84
*M. adhaerens*, axenic	1.8 ± 0.2	1.3 ± 0.1	0
*V. thalassae*, axenic	1.5 ± 0.1	1.4 ± 0.5	0
*P. carrageenovora*, axenic	1.6 ± 0.4	1.2 ± 0.3	0
*M. polymorphus* + *M. adhaerens*	*M. polymorphus*: 0.5 ± 0.08	*M. polymorphus*: 0.3 ± 0.09	**6.0** ± **2.83**
	*M. adhaerens*: 1.5 ± 0.1	*M. adhaerens*: 1.9^a^	
*M. polymorphus* + *V. thalassae*	*M. polymorphus*: 0.6 ± 0.2	*M. polymorphus*: 0.5 ± 0.1	0
	*V. thalassae*: 2.4 ± 0.05	*V. thalassae*: 2.7 ± 0.3	
*M. polymorphus* + *P. carrageenovora*	*M. polymorphus*: 0.5 ± 0.03	*M. polymorphus*: 0.4 ± 0.03	0
	*P. carrageenovora*: 2.3 ± 0.3	*P. carrageenovora*: 2.4 ± 0.4	
*M. polymorphus* + 2.5 µm beads	*M. polymorphus*: 0.8 ± 0.1	*M. polymorphus*: 0.6 ± 0.02	1.50 ± 2.12
	Beads: 1.2 ± 0.07	Beads: 1.1 ± 0.02	
*M. adhaerens* + 2.5 µm beads	*M. adhaerens*: 2.2 ± 0.2	*M. adhaerens*: 1.8 ± 0.6	0
	Beads: 0.6 ± 0.1	Beads: 0.5 ± 0.07	
*V. thalassae* + 2.5 µm beads	*V. thalassae*: 1.6 ± 0.2	*V. thalassae*: 1.3 ± 0.05	0
	Beads: 0.7 ± 0.2	Beads: 0.5 ± 0.01	
*P. carrageenovora* + 2.5 µm beads	*P. carrageenovora*: 1.5 ± 0.08	*P. carrageenovora*: 1.4 ± 0.003	0
	Beads: 0.2 ± 0.07	Beads: 0.1 ± 0.03	

Values are the means ± standard deviations of duplicate tanks (cell and aggregate abundance) or aggregates (all other parameters). Values in bold are significantly different to axenic *M. polymorphus*.

^a^Loss of replicate sample.

**Table 4:** Abundance, sinking velocity, and size of aggregates formed in roller tank experiments inoculated to higher total cell abundances (10^5^ cells mL^−1^).

Sinking aggregate formation in roller tanks End of bloom and sedimentation					
Treatment	Background cell abundance (×10^5^ mL^−1^)		Aggregate abundance (per tank)	Sinking velocity (m d^−1^)	ESD (mm)
	Before rotation	After rotation			
*M. polymorphus*, axenic	2.1 ± 0.4	1.6 ± 0.2	1.67 ± 2.89	304 ± 105 *n* = 5	1.9 ± 0.25 *n* = 3
*M. adhaerens*, axenic	1.6 ± 0.06	1.5 ± 0.6	0	-	-
*M. polymorphus* + *M. adhaerens*	*M. polymorphus*: 1.8 ± 0.5	*M. polymorphus*: 1.1 ± 0.1	**13.70** ± **4.04**	-	-
	*M. adhaerens*: 2.4 ± 0.1	*M. adhaerens*: 2.7 ± 0.3			

Values are the means ± standard deviations of triplicate tanks (cell and aggregate abundance) or aggregates (all other parameters). Values in bold are significantly different to axenic *M. polymorphus*.

- No data.

## Discussion

Our results support the hypothesis that the marine nano-diatom *Minutocellus polymorphus* forms fast-sinking (up to 485 m d^−1^) aggregates within a matrix of TEP. The sinking rates of these aggregates were comparable to those formed by bloom-forming diatom genera that aggregate readily in laboratory conditions and are associated with events of high POC flux, such as C*haetoceros* spp., *Navicula* spp., and *Skeletonema* spp., with sinking rates ranging between ca. 100 and 400 m d^−1^ (17, 36, 37). Our findings support that cell aggregation is the likely mechanism leading to the observations of nano- and pico-sized diatoms, such as *M. polymorphus*, in sediment traps deployed in the Sargasso Sea [5, 10] and in the Mediterranean [8].

Despite the lower abundance of cells in roller tanks compared to other aggregation studies (10^3^ in our lower cell abundance experiments vs. up to 10^6^ cells mL^−1^; 20, 36–38), *M. polymorphus* formed visible, sinking aggregates. As aggregation is highly dependent on the number of surrounding particles and the probability with which they adhere after collision (stickiness; α) [13], a likely explanation for the formation of aggregates despite relatively low particle numbers is the high α of *M. polymorphus* due to the production of TEP. Cell volume-normalized TEP concentrations in *M. polymorphus* cultures were two times higher than in cultures of other diatom species known to readily produce TEP and sticky particulate mucus [15] (Table S1). These diatoms are known to contribute appreciably to phytoplankton blooms and therefore contribute to events of high POC flux [39, 40]. Likewise, *M. polymorphus* bloom at cell abundances of up to 10^3^ cells mL^−1^ and predominate in sediment trap material relative to the populations in the euphotic zone [5, 10], the latter possibly being a result of the formation of TEP-rich, yet fast-sinking aggregates as seen in our study. The sinking rates for *M. polymorphus* aggregates shown herein would allow for cells within aggregates to escape grazing and remineralization through the microbial loop in the water column, contrary to the common assertion that they are entirely remineralized because of their small size and low sinking rates. In nature, the formation of TEP-rich, sticky aggregates by *M. polymorphus* would likely scavenge additional ballasting material, such as biogenic or lithogenic minerals [41], further enhancing the sinking velocities of their aggregates.

We also found that the aggregation and TEP production of *M. polymorphus* is differentially enhanced by the species of bacteria present. *V. thalassae* and *P. carrageenovora* were pre-selected based on indicator species analyses [42, 43] performed on 16S rRNA libraries of bacterial communities on sinking aggregates collected from the mesopelagic of the Sargasso Sea, whereby *Vibrio* spp. and *Pseudoalteromonas* spp. were specific to sinking particles [10]. A third species, *Marinobacter adhaerens* HP15 [26, 44], was selected based on their propensity to attach to and enhance the aggregation of the diatom *Thalassiosira weissflogii* [19]. When co-cultured with *M. polymorphus*, *M. adhaerens* HP15 caused a significant increase in the formation of suspended micro-aggregates (5–60 µm; Table 2) and of sinking aggregates (Fig. 4; Tables 3 and 4), similar to its enhancement of aggregation in the diatom *Thalassiosira weissflogii* found by Gärdes et al. [19]. However, co-cultures with *M. adhaerens* did not produce more TEP than axenic treatments, in contrast with the increase of TEP in co-cultures with *T. weissflogii* as reported by Gärdes et al. [45]. It is possible that TEP and/or its precursors were simultaneously being consumed and exuded by *M. adhaerens*—ultimately leading to a pool of bacteria-derived exopolymeric substances and/or TEP that are stickier than those derived from the diatoms [46–48], and to the observed enhanced formation of suspended micro-aggregates without increased TEP concentrations (Fig. 1c, d; Table 2). Elucidating the compositional differences of exopolymeric substances and investigating the production of other exopolymers that could also play a role in aggregation would help explain the mechanisms for the aggregation observed between *M. polymorphus* and *M. adhaerens*. Contrastingly, the addition of *P. carrageenovora* (a member of the Gammaproteobacteria) did not lead to enhanced aggregation in roller tanks (Fig. 4; Table 3), but to an enhanced volume concentration of suspended micro-aggregates (Fig. 2d, Table 2) along with lower cell-normalized TEP concentrations compared to axenic diatom cultures (Table 2). Members of the Gammaproteobacteria are able to hydrolyze complex polysaccharides [49]. Specifically, *Pseudoalteromonas* spp. readily attach to [50] and degrade a wide-range of phytoplankton-derived polysaccharides, including the relatively-recalcitrant fucose-containing sulphated polysaccharides [51, 52], by utilizing many different carbohydrate-active enzymes [51]. Therefore, *M. polymorphus-*derived TEP was likely being consumed by *P. carrageenovora* which is also supported by the decrease in TEP concentrations that occurred parallel to increases in *P. carrageenovora* cell abundances (Fig. 2b, e). As known colonizers and degraders of marine particles and phytoplankton-derived organic matter [51, 53, 54], the greater volume concentration of suspended micro-aggregates in *P. carrageenovora* treatments during the stationary and decline phases of the co-cultured *M. polymorphus* (Table 2) may be a result of aggregation of *P. carrageenovora* cells around free TEP and/or around *M. polymorphus* aggregates in order to consume the diatom-derived TEP (Figs. 2d and 3h). Lastly, no enhanced aggregation was observed with the addition of *V. thalassae* to *M. polymorphus* in roller tanks or cultures; however, TEP was significantly higher, coinciding with an increase in bacterial abundances. *V. thalassae* may have produced TEP or enhanced the production of TEP by *M. polymorphus* within co-cultures, but the lack of increased suspended micro-aggregates indicates a decoupling with TEP production. This finding also suggests other mechanisms of bacteria-mediated aggregation besides influencing TEP production and/or modifying its precursors, as was found with *M. adhaerens*. These results show that various TEP and aggregation-influencing interactions may occur within a natural bacterial community. In the environment, these interactions would be more complex as a result of interactions between diverse bacterial and phytoplankton communities, their grazers, as well as the influence of nutrient availability and other environmental factors [45] on these interactions.

Cell biovolume-normalized TEP concentrations of *M*. *polymorphus* cultures were an order of magnitude lower than those of *Synechococcus* sp. as found by Cruz and Neuer [20] (Table S1). Despite these lower TEP concentrations, *M. polymorphus* formed sinking aggregates in roller tanks, in contrast to *Synechococcus*, which do not form aggregates in axenic conditions unless lithogenic material is added [20]. One possible explanation for this is *M. polymorphus’* larger effective size due to the formation of long chains, in contrast to the unicellular lifestyle of *Synechococcus*. The presence of larger particles with higher excess densities within *M. polymorphus* roller tanks allow for greater collision rates and thus facilitate aggregation. The greater aggregation in *M. polymorphus* is also seen in cultures, whereby suspended micro-aggregate volume concentrations were approximately five times higher (39 ± 19 × 10^6^ µm^3^ mL^−1^ in axenic cultures during the exponential phase) than in *Synechococcus* [20] (7.6 ± 0.41 × 10^6^ µm^3^ mL^−1^ in axenic cultures), likely due to the presence of aggregated diatom chains (Fig. 3). Additionally, TEP produced by *M. polymorphus* may be stickier [47, 48] than *Synechococcus*-derived TEP, further increasing the chances of aggregation of *M. polymorphus* chains.

An alternative mechanism explaining the presence of *M. polymorphus* in 18S rRNA datasets from large size-fractionated seawater [9, 55] and sediment trap samples [5, 10] is their endosymbiotic affiliation with foraminifera (usually > 100 µm) [56]. Foraminifera are common components of sinking material collected within sediment traps and are therefore significant contributors to particle fluxes [57, 58], as their large size and CaCO_3_-derived shells allow for high sinking rates. The assimilation of *M. polymorphus* as endosymbionts by foraminifera could serve as a mechanism transporting them to depth, similar to the export of *Phaeocystis* spp. as endosymbionts of Acantharia [59] and the diazotrophic cyanobacteria *Richelia* spp. as endosymbionts of rhizosolenid diatoms [40]. Additionally, it is possible that aggregation, as seen in our study, allows for *M. polymorphus* cells and chains to become readily available as prey for zooplankton, with subsequent export in the form of fecal pellets [60, 61]. Nevertheless, our observations give further evidence to the underestimation of the contribution of these diatom genera to particle fluxes.

Our study is the first to show the potential for nano-sized eukaryotic phytoplankton to form sinking aggregates and produce TEP and provides evidence that this aggregation is differentially influenced by the ambient bacterial community. We also show that bacteria-mediated aggregation of phytoplankton is likely influenced by other exudates other than the TEP, which calls for the investigation of other potential aggregation-enhancing organic compounds. Our results contribute to the understanding of the mechanisms by which eukaryotic pico- and nanophytoplankton escape remineralization in the euphotic zone and are exported to the deep ocean.

## Supplementary information

Supplementary Information

## References

[1] Nelson DM, Tréguer P, Brzezinski MA, Leynaert A, Quéguiner B (1995). Production and dissolution of biogenic silica in the ocean: revised global estimates, comparison with regional data and relationship to biogenic sedimentation. Global Biogeochem Cycles.

[2] Jin X, Gruber N, Dune JP, Sarmiento JL, Armstrong RA (2006). Diagnosing the contributions of phytoplankton functional groups to the production and export of particulate organic carbon, CaCO_3_, and opal from global nutrient and alkalinity distributions. Global Biogeochem Cycles.

[3] Tréguer P, Bowler C, Moriceau B, Dutkiewicz S, Gehlen M, Aumont O (2018). Influence of diatom diversity on the ocean biological carbon pump. Nat Geosci..

[4] Michaels AF, Silver MW (1988). Primary production, sinking fluxes and the microbial food web. Deep Sea Res. Part I Ocean Res Pap.

[5] Amacher J, Neuer S, Lomas M (2013). DNA-based molecular fingerprinting of eukaryotic protists and cyanobacteria contributing to sinking particle flux at the Bermuda Atlantic time-series study. Deep Res Part II Top Stud Oceanogr..

[6] Brew HS, Moran SB, Lomas MW, Burd AB (2009). Plankton community composition, organic carbon and thorium-234 particle size distributions, and particle export in the Sargasso Sea. J Mar Res..

[7] Richardson TL (2019). Mechanisms and pathways of small-phytoplankton export from the surface ocean. Ann Rev Mar Sci.

[8] Leblanc K, Quéguiner B, Diaz F, Cornet V, Michel-Rodriguez M, Durrieu de Madron X (2018). Nanoplanktonic diatoms are globally overlooked but play a role in spring blooms and carbon export. Nat Commun..

[9] Vargas CDE, Audic S, Henry N, Decelle J, Mahé F, Logares R (2015). Eukaryotic plankton diversity in the sunlit ocean. Science.

[10] Cruz BN, Brozak S, Neuer S (2021). Microscopy and DNA-based characterization of sinking particles at the Bermuda Atlantic Time-series Study station point to zooplankton mediation of particle flux. Limnol. Oceanogr..

[11] Bolaños LM, Karp-Boss L, Choi CJ, Worden AZ, Graff JR, Haëntjens N, et al. Small phytoplankton dominate western North Atlantic biomass. *ISME J*. 2020;14:1663–74. 10.1038/s41396-020-0636-0 PMC7305139 32231247

[12] Waite AM, Safi KA, Hall JA, Nodder SD (2000). Mass sedimentation of picoplankton embedded in organic aggregates. Limnol Oceanogr..

[13] Jackson GA (1990). A model for the formation of marine algal flocks by physical coagulation processes. Deep Res..

[14] Passow U, Alldredge AL (1995). Aggregation of a diatom bloom in a mesocosm: the role of transparent exopolymer particles (TEP). Deep Sea Res Part II Top Stud Oceanogr.

[15] Kiørboe T, Hansen JLS (1993). Phytoplankton aggregate formation: observations of patterns and mechanisms of cell sticking and the significance of exopolymeric material. J Plankton Res..

[16] Alldredge AL, Passow U, Logan BE (1993). The abundance and significance of a class of large, transparent organic particles in the ocean. Deep Sea Res I.

[17] Engel A, Schartau M (1999). Influence of transparent exopolymer particles (TEP) on sinking velocity of *Nitzschia closterium* aggregates. Mar Ecol Prog Ser..

[18] Passow U (2002). Transparent exopolymer particles (TEP) in aquatic environments. Prog Oceanogr..

[19] Gärdes A, Iversen MH, Grossart H-P, Passow U, Ullrich MS (2011). Diatom-associated bacteria are required for aggregation of *Thalassiosira weissflogii*. ISME J.

[20] Cruz BN, Neuer S (2019). Heterotrophic bacteria enhance the aggregation of the marine picocyanobacteria *Prochlorococcus* and *Synechococcus*. Front. Microbiol.

[21] Tran NAT, Tamburic B, Evenhuis CR, Seymour JR (2020). Bacteria-mediated aggregation of the marine phytoplankton *Thalassiosira weissflogii* and *Nannochloropsis oceanica*. J. Appl. Phycol..

[22] Roux P, Siano R, Collin K, Bilien G, Sinquin C, Marchand L (2021). Bacteria enhance the production of extracellular polymeric substances by the green dinoflagellate *Lepidodinium chlorophorum*. Sci Rep..

[23] Radić T, Ivančić I, Fuks D, Radić J (2006). Marine bacterioplankton production of polysaccharidic and proteinaceous particles under different nutrient regimes. FEMS Microbiol Ecol.

[24] Grossart H-P, Czub G, Simon M (2006). Algae-bacteria interactions and their effects on aggregation and organic matter flux in the sea. Environ Microbiol..

[25] Rochelle-Newall EJ, Mari X, Pringault O (2010). Sticking properties of transparent exopolymeric particles (TEP) during aging and biodegradation. J Plankton Res..

[26] Grossart H-P, Schlingloff A, Bernard M, Simon M, Brinkhoff T (2004). Antagonistic activity of bacteria isolated from organic aggregates of the German Wadden Sea. FEMS Microbiol Ecol.

[27] Hasle GR, Stosch vonHA, Syvertsen EE (1983). Cymatosiraceae, a new diatom family. Bacillaria.

[28] Guillard RRL, Hargraves PE (1993). *Stichochrysis immobilis* is a diatom, not a chrysophyte. Phycologia.

[29] Kester DR, Duedall IW, Connors DN, Pytkowicz RM (1967). Preparation of artificial seawater. Limnol Oceanogr..

[30] Passow U, Alldredge AL (1995). A dye-binding assay for the spectrophotometric measurement of transparent exopolymer particles (TEP). Limnol Oceanogr..

[31] Bittar TB, Passow U, Hamaraty L, Bidle KD, Harvey EL (2018). An updated method for the calibration of transparent exopolymer particle measurements. Limnol Oceanogr Methods.

[32] Iuculano F, Mazuecos IP, Reche I, Agustí S (2017). *Prochlorococcus* as a possible source for transparent exopolymer particles (TEP). Front Microbiol..

[33] Shanks AL, Edmondson EW (1989). Laboratory-made artificial marine snow: a biological model of the real thing. Mar Biol..

[34] Ploug H, Kaufmann A, Wolf-gladrow D, Passow U (2010). A novel method to measure particle sinking velocity in vitro, and its comparison to three other in vitro methods. Limnol Oceanogr Methods.

[35] R Development Core Team. (2011). R: a language and environment for statistical computing.

[36] Iversen MH, Ploug H (2010). Ballast minerals and the sinking carbon flux in the ocean: carbon-specific respiration rates and sinking velocity of marine snow aggregates. Biogeosciences.

[37] Zetsche EM, Larsson AI, Iversen MH, Ploug H (2020). Flow and diffusion around and within diatom aggregates: effects of aggregate composition and shape. Limnol Oceanogr..

[38] Seebah S, Fairfield C, Ullrich MS, Passow U (2014). Aggregation and sedimentation of *Thalassiosira weissflogii* (diatom) in a warmer and more acidified future ocean. PLoS One.

[39] Preston CM, Durkin CA & Yamahara KM. DNA metabarcoding reveals organisms contributing to particulate matter flux to abyssal depths in the North East Pacific ocean. *Deep Res Part II* 2020;173:104708.

[40] Poff KE, Leu AO, Eppley JM, Karl DM, DeLong EF (2021). Microbial dynamics of elevated carbon flux in the open ocean’s abyss. Proc Natl Acad Sci USA.

[41] van der Jagt H, Friese C, Stuut JBW, Fischer G, Iversen MH (2018). The ballasting effect of Saharan dust deposition on aggregate dynamics and carbon export: aggregation, settling, and scavenging potential of marine snow. Limnol Oceanogr..

[42] Dufrêne M, Legendre P (1997). Species assemblages and indicator species: the need for a flexible asymmetrical approach. Ecol Monogr..

[43] De Cáceres M, Legendre P, Moretti M (2010). Improving indicator species analysis by combining groups of sites. Oikos.

[44] Kaeppel EC, Gärdes A, Seebah S, Grossart HP, Ullrich MS (2012). *Marinobacter adhaerens* sp. nov., isolated from marine aggregates formed with the diatom *Thalassiosira weissflogii*. Int J Syst Evol Microbiol.

[45] Gärdes A, Ramaye Y, Grossart HP, Passow U, Ullrich MS (2012). Effects of *Marinobacter adhaerens* HP15 on polymer exudation by *Thalassiosira weissflogii* at different N:P ratios. Mar Ecol Prog Ser..

[46] Li S, Winters H, Villacorte LO, Ekowati Y, Emwas AM, Kennedy MD (2015). Compositional similarities and differences between transparent exopolymer particles (TEPs) from two marine bacteria and two marine algae: Significance to surface biofouling. Mar Chem..

[47] Santschi PH, Xu C, Schwehr KA, Lin P, Sun L, Chin WC (2020). Can the protein/carbohydrate (P/C) ratio of exopolymeric substances (EPS) be used as a proxy for their ‘stickiness’ and aggregation propensity?. Mar Chem..

[48] Chen CS, Shiu RF, Hsieh YY, Xu C, Vazquez CI, Cui Y (2021). Stickiness of extracellular polymeric substances on different surfaces via magnetic tweezers. Sci Total Environ..

[49] Sarmento H, Morana C, Gasol JM (2016). Bacterioplankton niche partitioning in the use of phytoplankton-derived dissolved organic carbon: quantity is more important than quality. ISME J.

[50] Yamada Y, Fukuda H, Tada Y, Kogure K, Nagata T (2016). Bacterial enhancement of gel particle coagulation in seawater. Aquat Microb Ecol..

[51] Gobet A, Barbeyron T, Matard-Mann M, Magdelenat G, Vallenet D, Duchaud E (2018). Evolutionary evidence of algal polysaccharide degradation acquisition by *Pseudoalteromonas carrageenovora* 9T to adapt to macroalgal niches. Front Microbiol..

[52] Vidal-Melgosa S, Sichert A, Francis BT, Bartosik D, Niggeman J, Wichels A (2021). Diatom fucan polysaccharide precipitates carbon during algal blooms. Nat Commun..

[53] Stocker R, Seymour JR, Samadani A, Hunt DE, Polz MF (2008). Rapid chemotactic response enables marine bacteria to exploit ephemeral microscale nutrient patches. Proc Natl Acad Sci USA.

[54] Seymour JR, Ahmed T, Durham WM, Stocker R (2010). Chemotactic response of marine bacteria to the extracellular products of *Synechococcus* and *Prochlorococcus*. Aquat Microbial Ecol.

[55] Guidi L, Chaffron S, Bittner L, Eveillard D, Larhlimi A, Roux S (2016). Plankton networks driving carbon export in the oligotrophic ocean. Nature.

[56] Schmidt C, Morard R, Romero O, Kucera M (2018). Diverse internal symbiont community in the endosymbiotic foraminifera *Pararotalia calcariformata*: Implications for symbiont shuffling under thermal stress. Front Microbiol..

[57] Caron DA, Michaels AF, Swanberg NR, Howse FA (1995). Primary productivity by symbiont-bearing planktonic sarcodines (Acantharia, Radiolaria, Foraminifera) in surface waters near Bermuda. J Plankton Res..

[58] Schiebel R (2002). Planktic foraminiferal sedimentation and the marine calcite budget. Glob Biogeochem Cycles.

[59] Decelle J, Probert I, Bittner L, Desdevises Y, Colin S, Vargas (2012). An original mode of symbiosis in open ocean plankton. Proc Natl Acad Sci USA.

[60] Wilson SE, Steinberg DK (2010). Autotrophic picoplankton in mesozooplankton guts: evidence of aggregate feeding in the mesopelagic zone and export of small phytoplankton. Mar Ecol Prog Ser.

[61] Stukel MR, Décima M, Selph KE, Taniguchi DAA, Landry MR (2013). The role of *Synechococcus* in vertical flux in the Costa Rica upwelling dome. Prog Oceanogr..

