# Supplementary material for: Particle-associated bacteria differentially influence the aggregation of the marine diatom Minutocellus polymorphus
Source: ISME Commun. 2022 Aug 18;2:73. doi: 10.1038/s43705-022-00146-z (PMC9723735; doi:10.1038/s43705-022-00146-z)
Supplement: Supplementary file 1 — Supplementary Information [file 43705_2022_146_MOESM1_ESM.docx]

**Supplementary Table. 1** Comparison of cell biovolume-normalized transparent exopolymeric particle (TEP) concentrations calculated in this study with those of xenic and axenic phytoplankton from the literature. All data are from batch cultures in exponential growth phase. Values from this study are the means ± standard deviations of triplicate treatments.

| **Species** | **Cell volume-normalized TEP concentration (**×**10^−9^ µg XG eq. µm^−3^)** | | **Study** |
| --- | --- | --- | --- |
|  | Xenic | Axenic |  |
| *Chaetoceros affinis* (CCMP 159) | 22 | - | Passow 2002 |
| *Coscinodiscus granii* | - | 341.6 ± 56.33 | Fukao et al. 2010 |
| *Rhizosolenia setigera* | - | 74.7 ± 8.63 |  |
| *Skeletonema* sp. | - | 68.4 ± 3.28 |  |
| *Synechococcus* sp. | 1,758 ± 278^c^ | 1,028 ± 337^c^ | Cruz and Neuer 2019 |
| *Prochlorococcus marinus* (MED4) | 28 ± 3^d^ | <1^d^ |  |
| *M. polymorphus* | - | 116 ± 37 | This study |
| *M. polymorphus* + *M. adhaerens* | 181 ± 18^e^ | - |  |
| *M. polymorphus + V. thalassae* | 118 ± 46^e^ |  |  |
| *M. polymorphus* + *P. carrageenovora* | 160 ± 58^e^ |  |  |

- : No data.

Cell volumes were calculated assuming the following diameters: a. 5 µm (Roscoff Culture Collection), b. 0.6 µm (Roscoff Culture Collection), c. 1 µm (NCMA), d. 0.8 µm (NCMA), e. 3 µm.

**Note.** All experimental culture media utilized by Passow (2002), Fukao et al. (2010), and this study are identical. While the media in Cruz et al. (2019) differ to the others in their trace metal and vitamin composition, the N:P ratios are the same. However, the light intensities in Passow (2002) and Fukao et al. (2010) were higher than in this study and Cruz et al. (2019), with Fukao et al. incubating diatoms at 150 µmol photons m^-2^ s^-1^, approximately 70 µmol photons m^-2^ s^-1^ higher than our incubations. Despite the higher light intensities in other studies, which would lead to increased TEP exudation as seen in Rabouille et al. (2017), our TEP concentrations were higher, giving more significance to the relatively higher concentrations seen in our *Minutocellus* cultures.

**References**

Cruz, B. N. & Neuer, S. Heterotrophic bacteria enhance the aggregation of the marine picocyanobacteria *Prochlorococcus* and *Synechococcus*. *Front. Microbiol.* **10**, 1–11 (2019).

Fukao, T., Kimoto, K. & Kotani, Y. Production of transparent exopolymer particles by four diatom species. *Fish. Sci.* **76**, 755–760 (2010).

Passow, U. Production of transparent exopolymer particles (TEP) by phyto- and bacterioplankton. *Mar. Ecol. Prog. Ser.* **236**, 1–12 (2002)

Rabouille, S., G. S. Cabral, and M. L. Pedrotti. 2017. Towards a carbon budget of the diazotrophic cyanobacterium *Crocosphaera*: Effect of irradiance. Mar. Ecol. Prog. Ser. 570: 29–40. doi:10.3354/meps12087
